# Abnormal brain oxygen homeostasis in an animal model of liver disease

**DOI:** 10.1016/j.jhepr.2022.100509

**Published:** 2022-05-24

**Authors:** Anna Hadjihambi, Cristina Cudalbu, Katarzyna Pierzchala, Dunja Simicic, Chris Donnelly, Christos Konstantinou, Nathan Davies, Abeba Habtesion, Alexander V. Gourine, Rajiv Jalan, Patrick S. Hosford

**Affiliations:** 1 UCL Institute for Liver and Digestive Health, Division of Medicine, UCL Medical School, Royal Free Hospital, Rowland Hill Street, London, UK; 2 Centre for Cardiovascular and Metabolic Neuroscience, Neuroscience, Physiology and Pharmacology, University College London, London, UK; 3 The Roger Williams Institute of Hepatology London, Foundation for Liver Research, London, UK; 4 Faculty of Life Sciences and Medicine, King’s College London, London, UK; 5 CIBM Center for Biomedical Imaging, Lausanne, Switzerland; 6 Animal Imaging and Technology, Ecole Polytechnique Fédérale de Lausanne (EPFL), Lausanne, Switzerland; 7 Laboratory of Functional and Metabolic Imaging, Ecole Polytechnique Fédérale de Lausanne (EPFL), Lausanne, Switzerland; 8 Institute of Sports Science and Department of Physiology, University of Lausanne, Lausanne, Switzerland; 9 William Harvey Research Institute, Barts and the London School of Medicine and Dentistry, London, UK; 10 European Foundation for the Study of Chronic Liver Failure

**Keywords:** Oxygen, Ornithine phenylacetate, Chronic liver disease, Hyperammonaemia, Phenylephrine, hepatic encephalopathy, Ala, alanine, AIT, Animal Imaging and Technology, ALT, alanine transaminase, Asc, ascorbate, Asp, aspartate, ATZ, acetazolamide, BDL, bile duct ligation, BOLD, blood oxygen level dependent, BP, blood pressure, CBF, cerebral blood flow, CIBM, Center for Biomedical Imaging, CLD, chronic liver disease, CMRO_2_, cerebral metabolic rate of oxygen, CNS, central nervous system, Cr, creatine, eNOS, endothelial nitric oxide synthase, EPFL, Ecole Polytechnique Fédérale de Lausanne, [^18^F]-FDG PET, [18F]-fluorodeoxyglucose positron emission tomography, fMRI, functional magnetic resonance imaging, GABA, γ-aminobutyric acid, Glc, glucose, Gln, glutamine, Glu, glutamate, GPC, glycerophosphocholine, GSH, glutathione, HE, hepatic encephalopathy, ^1^H-MRS, proton magnetic resonance spectroscopy, Ins, myo-inositol, Lac, lactate, MAP, mean arterial pressure, mHE, minimal HE, NAA, N acetylaspartate, NO, nitric oxide, OP, ornithine phenylacetate, PCho, phosphocholine, PCr, phosphocreatine, pCO_2_, partial pressure of carbon dioxide, PE, phenylephrine, pO_2_, partial pressure of oxygen, SPECIAL, spin echo full intensity acquired localised, Tau, taurine, tCho, total choline, tCr, total creatine, TE, echo time, VOI, volume of interest

## Abstract

**Background & Aims:**

Increased plasma ammonia concentration and consequent disruption of brain energy metabolism could underpin the pathogenesis of hepatic encephalopathy (HE). Brain energy homeostasis relies on effective maintenance of brain oxygenation, and dysregulation impairs neuronal function leading to cognitive impairment. We hypothesised that HE is associated with reduced brain oxygenation and we explored the potential role of ammonia as an underlying pathophysiological factor.

**Methods:**

In a rat model of chronic liver disease with minimal HE (mHE; bile duct ligation [BDL]), brain tissue oxygen measurement, and proton magnetic resonance spectroscopy were used to investigate how hyperammonaemia impacts oxygenation and metabolic substrate availability in the central nervous system. Ornithine phenylacetate (OP, OCR-002; Ocera Therapeutics, CA, USA) was used as an experimental treatment to reduce plasma ammonia concentration.

**Results:**

In BDL animals, glucose, lactate, and tissue oxygen concentration in the cerebral cortex were significantly lower than those in sham-operated controls. OP treatment corrected the hyperammonaemia and restored brain tissue oxygen. Although BDL animals were hypotensive, cortical tissue oxygen concentration was significantly improved by treatments that increased arterial blood pressure. Cerebrovascular reactivity to exogenously applied CO_2_ was found to be normal in BDL animals.

**Conclusions:**

These data suggest that hyperammonaemia significantly decreases cortical oxygenation, potentially compromising brain energy metabolism. These findings have potential clinical implications for the treatment of patients with mHE.

**Lay summary:**

Brain dysfunction is a serious complication of cirrhosis and affects approximately 30% of these patients; however, its treatment continues to be an unmet clinical need. This study shows that oxygen concentration in the brain of an animal model of cirrhosis is markedly reduced. Low arterial blood pressure and increased ammonia (a neurotoxin that accumulates in patients with liver failure) are shown to be the main underlying causes. Experimental correction of these abnormalities restored oxygen concentration in the brain, suggesting potential therapeutic avenues to explore.

## Highlights

• Using a rat model, we showed that HE is associated with reduced brain tissue oxygen, glucose and lactate content.
• Cerebrovascular dilating agents and ammonia-lowering treatment reduced the effect on brain oxygenation.
• Ammonia could therefore act by increasing central vascular tone.
• Cerebrovascular tone could be a potential therapeutic target alongside ammonia-lowering strategies.

## Graphical abstract

**Figure:**
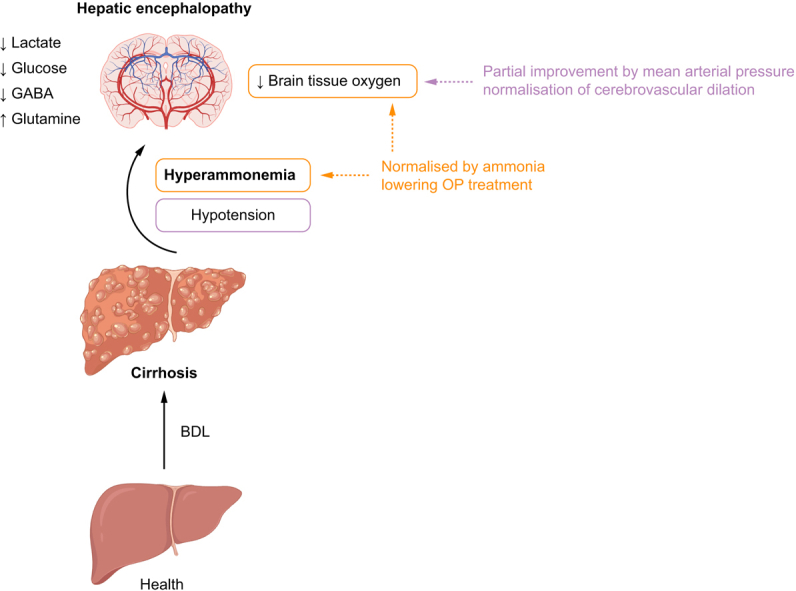

## Introduction

Hepatic encephalopathy (HE) in chronic liver disease (CLD) is characterised by a spectrum of neuropsychiatric symptoms that include impairment of cognitive function.^1^^,^^2^ This is a serious but potentially reversible condition that severely limits the patient’s quality of life and long-term prognosis. HE can progress quickly, resulting in coma with mortality rates of up to 50%^3^ without liver transplantation. For some patients, despite successful transplantation, the neuropsychiatric symptoms can persist indefinitely.^4^ Blood ammonia concentration is one of the main mechanisms thought to underlie the development of HE^5^ and is an important therapeutic target.^6^ However, the exact mechanism of how hyperammonaemia leads to this complex neuropsychiatric syndrome is still unclear. What is known is that excess ammonia in the central nervous system (CNS) impacts both astrocytic and neuronal function to impair cognitive processing in a graded, progressive fashion.^7^ A more granular understanding of the pathophysiology could inform better treatment regimens and identify additional drug targets.

Cognitive impairment seen in HE may be the result of several related factors, including altered glutamatergic^8^ and GABAergic neurotransmission,^9^ as well as (early) compromised brain energy metabolism,[10], [11], [12] all of which are affected by or correlated with hyperammonaemia. The latter will impair all aspects of brain function as neurotransmission is a particularly metabolically demanding activity.^13^ A characteristic decline of whole-brain oxidative metabolism has been seen in patients with HE, which implicates changes of neurons and their energy turnover, rather than a malfunction of oxidative metabolism in astrocytes.^14^ Animal models using bile duct ligation (BDL)-induced elevation in ammonia have reported mitochondrial dysfunction, a reduction in both mitochondrial membrane potential and respiratory chain enzymes, and swelling of mitochondria.^15^ This culminates in impaired ATP generation and oxidative stress, which in turn leads to compromised brain energy metabolism.^11^^,^^16^

Oxygen is the key metabolic substrate within the CNS, but only a 1-s buffer in supply is continuously maintained. It is therefore necessary to tightly control delivery of this resource, and mechanisms have evolved to closely regulate blood flow to match oxygen supply with demand.^17^^,^^18^ Long-term impairment of cerebral blood flow (CBF) control and therefore oxygen delivery has been linked to the development and/or progression of cognitive impairment during ageing and Alzheimer’s disease.^19^^,^^20^ Similarly, acute impairment can have long-term consequences on neurological function.^21^ In patients with liver cirrhosis and HE, cerebral metabolic rate of oxygen (CMRO_2_) and CBF are decreased when compared to those patients with cirrhosis but without HE and also to healthy controls.^14^^,^^22^^,^^23^ It has not yet been possible to determine if these derangements are associated with brain hypoxia and whether hyperammonaemia contributes to this reduction.^24^

In this study, we hypothesised that HE is associated with brain hypoxia as a consequence of the high concentrations of circulating ammonia. Using the BDL rat model of CLD with minimal HE (mHE), we investigated the mechanism of HE-related low brain oxygen concentration by manipulating peripheral and cerebral perfusion. Additionally, we sought to clarify the role of ammonia in altering brain oxygenation during HE using the drug ornithine phenylacetate (OP, OCR-002; Ocera Therapeutics, CA, USA), which is known to reduce plasma and brain ammonia concentrations.^25^

## Materials and methods

All experiments were performed in accordance with the European Commission Directive 2010/63 (European Convention for the Protection of Vertebrate Animals used for Experimental and Other Scientific Purposes) and the UK Animals in Scientific Procedures Act 1986 (amended 2012), with project approval by the Institutional Animal Welfare and Ethical Review Board. All experiments were designed and reported in adherence to ARRIVE guidelines.^26^ Some experiments were performed in collaboration with the Center for Biomedical Imaging (CIBM), MRI Ecole Polytechnique Fédérale de Lausanne (EPFL) section, Animal Imaging and Technology (AIT), Lausanne, Switzerland, owing to the availability of proton magnetic resonance spectroscopy (^1^H-MRS) and were approved by the Committee on Animal Experimentation for the Canton of Vaud, Switzerland (VD3022.1). In both cases, experimental subjects were obtained from a commercial supplier, Charles Rivers Laboratories, Inc. Animals were group-housed in individually ventilated cages, enriched with rails and cardboard tubes, in a room of 20–22°C, relative moisture 50–60%, and 12-h light–dark cycle (light 7 am to 7 pm).

### Animal model of HE

HE in experimental animals was induced by BDL procedure as described previously.^10^^,^^27^ Briefly, under surgical anaesthesia (5% isoflurane in oxygen for induction and 2% isoflurane in air for maintenance), rats underwent triple ligation of the bile duct via a small laparotomy to induce advanced chronic liver injury. Control groups underwent a sham surgical procedure where the bile duct was exposed for equal time, before closure of the incision. Body temperature was monitored via a rectal probe and maintained at 37 ± 0.5°C with a Homeothermic Blanket Control Unit (Harvard). At the end of the experiments, blood was collected from the left ventricle of the heart under anaesthesia, and biochemical measurements were performed using a Cobas Integra II system (Roche Diagnostics) with plasma or PocketChem™ (BA PA-4140) with fresh blood (Table S1). Plasma bilirubin was measured using a Cobas Integra II system (Roche Diagnostics) or a Reflotron® Plus system (F. Hoffmann-La Roche Ltd) as indicated in Table S1.

**Fig. 1:**
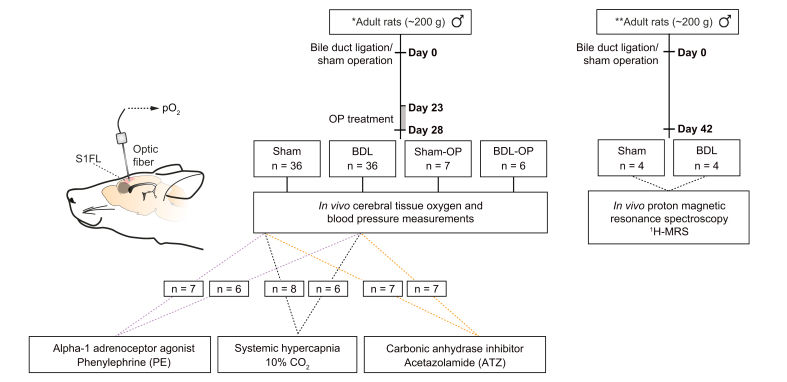
Study design overview. Schematic depicting the study design overview and animal number allocation. ∗Sprague Dawley rats, ∗∗Wistar rats. ^1^H-MRS, proton magnetic resonance spectroscopy; ATZ, acetazolamide; BDL, bile duct ligation; OP, ornithine phenylacetate; PE, phenylephrine; pO_2_, partial pressure of oxygen.

Brain tissue partial pressure of oxygen (pO_2_) measurements were performed in Sprague Dawley rats at 28 days post-surgery. ^1^H-MRS experiments were performed in Wistar rats at 42 days post-surgery, as previous studies have shown slower progression of liver disease development.^10^^,^^28^ Despite the difference in strain and duration post-surgery, the selected time points have previously been defined as the time required for each animal model to develop similar degree of severe fibrosis with manifestation of severe cholestasis, portal hypertension, and cerebral dysfunction,^10^^,^^29^ as well as similar ammonium and bilirubin concentrations (Table S1). The study overview and experimental design is schematised in Fig. 1.

### OP treatment

Combined doses of l-ornithine and phenylacetate (0.3 g/kg; OP) were given twice daily, by i.p. injections, 23 days after the surgery, ∼7 h apart for 5 days. This dosing regime has previously been shown to reduce plasma ammonia concentration by ∼50%.^30^ The rats were studied on day 28 after the BDL surgery, within 3 h of the last OP injection.

### Brain tissue oxygen measurements

Brain tissue oxygen was measured *in vivo* in BDL and sham-operated animals (Fig. 1). Anaesthesia was induced by isoflurane as stated above and maintained with α-chloralose (100 mg/kg, i.v.). Supplementary doses of α-chloralose (10–20 mg/kg, i.v.) were given as required. The depth of anaesthesia was assessed by the stability of cardiovascular and respiratory variables being recorded. The right femoral artery was cannulated for the measurement of blood pressure (BP) and for sampling arterial blood for analysis of pH and blood gases. Samples were collected at regular intervals and analysed using a pH/blood gas analyser (Siemens Rapidlab 248; Siemens Healthcare, Sudbury, UK). Blood gases and pH were maintained within the physiological range (pO_2_ 100–120 mmHg, partial pressure of carbon dioxide [pCO_2_] 30–40 mmHg, pH 7.35–7.40, and calculated bicarbonate between 22 and 26 meq/L) by adjusting the rate and/or stroke volume of the ventilator and by supplementary oxygen in the inspired room air. Body temperature was monitored via a rectal probe and maintained at 37 ± 0.5°C using a Homeothermic Blanket Control Unit (Harvard). BP was measured using a pressure transducer (Neurolog, Digitimer, UK), and heart rate was derived electronically from the BP signal.

Animals were placed in a stereotaxic frame, and a limited craniotomy was performed to access the somatosensory (forelimb) region of the cortex (S1FL ∼0.5 mm below the cortical surface; Fig. 1). pO_2_ was monitored using optical fluorescence technology that allows real-time detection of pO_2_ *in vivo* (OxyliteTM, Oxford Optronics), as previously described.^31^^,^^32^ Following the insertion of the sensor, the craniotomy was sealed from the air with petroleum jelly, preventing diffusion of ambient oxygen. Following a 15-min recovery period, parenchymal pO_2_ sampling was started until a stable reading was achieved.

### Pharmacological and blood gas manipulations

To investigate cerebrovascular reactivity and the role of peripheral and cerebral perfusion in altering cerebral cortical oxygen concentration, pharmacological and blood gas manipulations were performed. Systemic hypercapnia was induced by switching the input to the ventilator from room air to a compressed gas source that comprised 21% O_2_ and 10% CO_2_, with the balance made of nitrogen. Animals were exposed to this gas mixture for a period of 5 min after baseline pO_2_ was recorded. In a separate group of animals, sham-operated and BDL subjects (Fig. 1) received the carbonic anhydrase inhibitor acetazolamide (ATZ^33^; 10 mg/kg, i.v.) dissolved in 100% DMSO (maximum volume 25 μl) after baseline pO_2_ was recorded. Peripheral vessel tone under anaesthesia was manipulated by infusion of the alpha1-adrenoceptor agonist phenylephrine (PE). PE was infused at a rate of 5–10 μg/min to maintain a mean arterial pressure (MAP) in the BDL subjects of ∼100 mmHg for a period of approximately 10 min.

### *In vivo*^1^H-MRS at 9.4 T

To investigate the characteristic metabolic changes known to occur in HE^7^ and validate our model, sham-operated and BDL rats (Fig. 1) were anaesthetised with isoflurane (5% for induction and 2% for maintenance in 50% air and 50% oxygen) and underwent ^1^H-MRS. ^1^H-MRS spectra were acquired on a 9.4 T system (Varian/Magnex Scientific) using the spin echo full intensity acquired localised (SPECIAL) sequence (echo time [TE] = 2.8 ms) as previously described.^10^ Volume of interest (VOI) was selected in S1 primary somatosensory cortex (1.3 × 2 × 3 mm^3^). LCModel was used for quantification using water as internal reference, allowing the quantification of a total of 18 metabolites. Body temperature of animals was monitored via a rectal probe and maintained at 37 ± 0.5°C by means of a heated MRI-compatible animal cradle.

### Data analysis and statistics

Physiological variables were digitised using a Power 1401 interface (CED) and stored on a PC for offline processing using Spike 2 software (CED). Statistical analysis was performed using GraphPad Prism (v9 for Mac, San Diego, CA, USA). Data are expressed as mean ± SEM. Differences were ascertained using the Kruskal–Wallis test followed by Dunn’s multiple-comparison *post hoc* test or a paired/unpaired *t* test and the Mann–Whitney *U* test, where appropriate. Differences with a *p* value of <0.05 were considered significant.

## Results

### Biochemistry

Compared with sham surgery, the BDL procedure resulted in a significant increase in plasma ammonia, alanine transaminase (ALT) and bilirubin (*p* <0.001), indicating impaired liver function, whereas albumin and total protein concentrations were significantly decreased (*p* <0.001). Treatment of BDL animals with OP lowered plasma ammonia concentration, which was similar to that measured in sham-operated animals (*p* = 0.3), but had no effect on other parameters; ALT, bilirubin, albumin, and total protein concentrations remained unchanged from the untreated BDL group. Plasma biochemistry and ammonia concentration data are summarised in Tables S1 and S2.

### Brain tissue pO_2_ and cerebrovascular CO_2_ reactivity

**Fig. 2:**
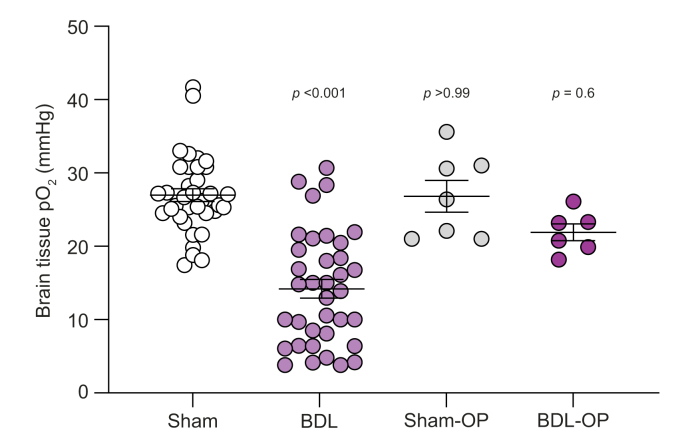
Cortical pO_2_ in an animal model of HE. Summary data illustrating basal pO_2_ in the somatosensory cortex of sham-operated, BDL, sham-OP, and BDL-OP-treated animals. Data are expressed as mean ± SEM and compared using the Kruskal–Wallis test followed by Dunn’s multiple-comparison *post hoc* test. Values of *p* indicate differences from sham-operated rats. BDL, bile duct ligation; OP, ornithine phenylacetate; pO_2_, partial pressure of oxygen.

Following placement of the oxygen sensor in the cerebral cortex (Fig. 1), blood pO_2_ and pCO_2_ were measured, and no significant differences were detected between groups (Table 1). Brain pO_2_ was obtained over a period of at least 5 min of stable recording. An average of this period revealed a significantly lower brain pO_2_ (BDL: 14 ± 1 mmHg, n = 36; sham-operated controls: 27 ± 1 mmHg, n = 36; *p* <0.001; Fig. 2).

**Table 1:** **Arterial blood pO**_**2**_**and pCO**_**2**_**in an animal model of HE, indicating no statistically significant differences between the groups**.

	Arterial blood pO_2_ (mmHg)	Arterial blood pCO_2_ (mmHg)
Sham	121 ± 2	32 ± 1
BDL	114 ± 3	33 ± 1
Sham-OP	116 ± 2	34 ± 2
BDL-OP	115 ± 5	31 ± 2

Data are expressed as mean ± SEM and compared using 1-way ANOVA. BDL, bile duct ligation; HE, hepatic encephalopathy; OP, ornithine phenylacetate; pCO_2_, partial pressure of carbon dioxide; pO_2_, partial pressure of oxygen.

To investigate the role of hyperammonaemia in brain oxygen impairment seen in our model of HE, we lowered ammonia by treating the BDL animals with OP, which had no significant effect on any other plasma biochemistry parameters (Table S1). OP treatment of BDL rats significantly improved brain pO_2_ (22 ± 1 mmHg, n = 6), increasing the oxygen concentration similar to that recorded in sham-operated (27 ± 1 mmHg; *p* = 0.6) and sham-OP rats (27 ± 2 mmHg, n = 7; *p* >0.1; Fig. 2).

**Fig. 3:**
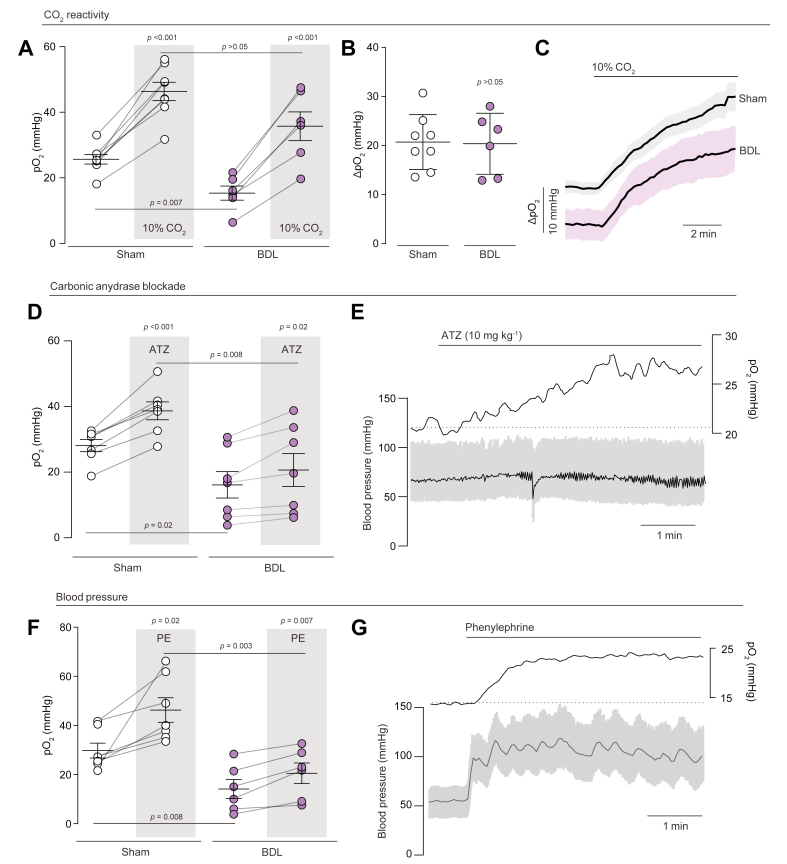
Effect of vessel tone manipulations on cortical pO_2_ in BDL animals. (A) Grouped data showing cortical peak pO_2_ changes from baseline in BDL and sham-operated control animals in response to hypercapnic acidosis (10% inspired CO_2_). (B) Grouped data comparing the relative change in pO_2_ in BDL and sham-operated control animals. Compared using the Mann–Whitney *U* test. (C) Time series of cortical pO_2_ changes in response to hypercapnic acidosis in BDL and sham-operated control animals. (D) Grouped data showing cortical peak pO_2_ changes from baseline in BDL and sham-operated control animals in response to carbonic anhydrase inhibition (ATZ, 10 mg/kg). (E) Example experimental trace showing the effect of ATZ on cortical pO_2_ and arterial blood pressure (black line indicates MAP) after BDL. (F) Grouped data showing cortical peak pO_2_ changes from baseline in BDL and sham-operated control animals in response to increased arterial blood pressure (PE infusion, 5–10 μg/min). (G) Example experimental trace showing the normalisation of arterial blood pressure with PE infusion after BDL and corresponding change in cortical pO_2_. All grouped data are expressed as mean ± SEM, using a paired sample *t* test when comparing an experimental manipulation within subject or an unpaired sample *t* test when comparing between groups of subjects, unless otherwise stated. ATZ, acetazolamide; BDL, bile duct ligation; MAP, mean arterial pressure; PE, phenylephrine; pO_2_, partial pressure of oxygen.

To evaluate the ability of cerebral vessels to respond to a known vasodilatory stimulus, hypercapnic acidosis was induced by changing the inspired gas mixture to include 10% CO_2_. Hypercapnic acidosis led to a significant increase in parenchymal pO_2_ from baseline in both BDL (*p* <0.001) and sham-operated rats (*p* <0.001; Fig. 3A and C), that is, 15 ± 2 to 36 ± 4 mmHg (n = 6) and 26 ± 1 to 46 ± 3 mmHg (n = 8), respectively. Despite the lower baseline pO_2_ (*p* = 0.007), CO_2_ reactivity was preserved in BDL animals, with an increase in pO_2_ (by 20 ± 3 mmHg, 80% increase) not significantly different to that observed in sham-operated animals (by 21 ± 2 mmHg, 132% increase, *p* = 0.9; Fig. 3B). This indicates that it is possible to restore brain oxygenation by cerebrovascular dilation as there was no difference (*p* = 0.1) between the peak tissue pO_2_ measured in BDL and sham-operated controls (Fig. 3A) during hypercapnic acidosis.

Brain pO_2_ could also be partially restored by pharmacological agents known to specifically dilate cerebral vasculature. The carbonic anhydrase inhibitor ATZ was chosen as it known to dilate the cerebrovasculature without significant effects on arterial blood pressure. Blockade of carbonic anhydrase causes an accumulation of extracellular protons to induce smooth muscle relaxation^34^^,^^35^ in the CNS. Doses of 10 mg/kg were found to significantly increase brain oxygenation in BDL animals, from 16 ± 4 to 21 ± 5 mmHg (n = 7, 28% increase, *p* = 0.02), although this was not as effective as in sham-operated animals where it increased from 28 ± 2 to 39 ± 3 mmHg (n = 7, 38% increase, *p* <0.001; Fig. 3D and E).

It has been previously reported that MAP is lower in conscious BDL animals.^36^ We confirmed this observation in the anaesthetised animals; BDL animals had a significantly lower MAP compared with sham-operated controls, with 60 ± 3 *vs*. 84 ± 8 mmHg, respectively (*p* = 0.04; Fig. S1). To control for the effects of a lower MAP on brain oxygenation, an infusion of the α1-adrenergic receptor agonist PE was used to normalise MAP to that of sham-operated animals (Fig. 3F and G). Increasing MAP in BDL animals significantly increased brain pO_2_ by 6 ± 1 mmHg from 14 ± 4 to 20 ± 4 mmHg (n = 6, 45% increase, *p* = 0.007). Inducing a corresponding change in MAP in sham-operated rats, brain oxygenation was increased by 16 ± 5 mmHg (n = 7, 55% increase, *p* = 0.02) compared with baseline. Interestingly, BDL animals receiving OP treatment did not show a significant improvement in MAP, with 69 ± 5 mmHg (n = 5) compared with 60 ± 3 mmHg in untreated animals (*p* = 0.15). Again, arterial blood pO_2_ and pCO_2_ were not different between groups (Table 1).

### *In vivo*^1^H-MRS at 9.4T

**Fig. 4:**
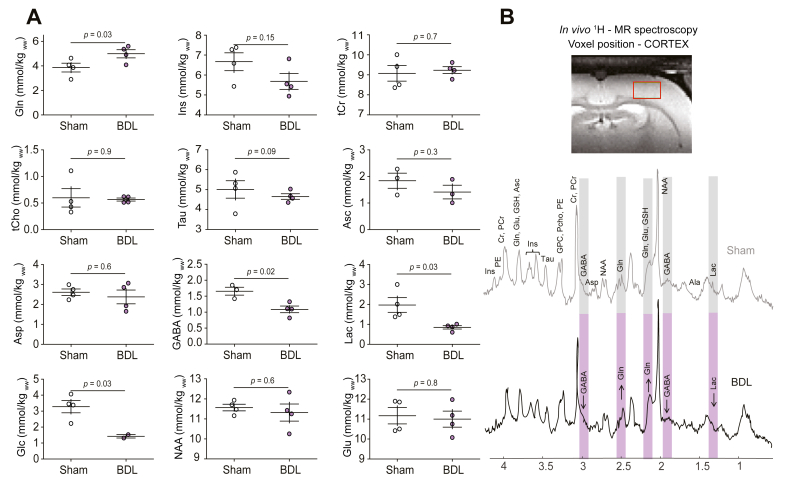
*In vivo* brain ^1^H-MRS results obtained in the somatosensory cortex of BDL and sham-operated animals. (A) Grouped data comparing changes of relevant metabolites and osmolytes in the somatosensory cortex of BDL and sham-operated control animals. (B) Representative ^1^H-MRS spectra measured in BDL and sham-operated animals with the corresponding voxel location (top panel). Metabolite changes are visual in the spectra (*i.e.* Gln, GABA, and Lac) and are highlighted in grey. Data are expressed as mean ± SEM and compared using an unpaired sample *t* test. ^1^H-MRS, proton magnetic resonance spectroscopy; Ala, alanine; Asc, ascorbate; Asp, aspartate; BDL, bile duct ligation; Cr, creatine; GABA, γ-aminobutyric acid; Glc, glucose; Gln, glutamine; Glu, glutamate; GPC, glycerophosphocholine; GSH, glutathione; Ins, myo-inositol; Lac, lactate; NAA, N acetylaspartate; PCho, phosphocholine; PCr, phosphocreatine; PE, phenylephrine; Tau, taurine; tCho, total choline; tCr, total creatine.

The characteristic metabolic pattern of chronic HE was observed in the somatosensory cortex of BDL rat (Fig. 4), characterised by a significant increase of glutamine (sham-operated: 3.8 ± 0.7, BDL: 5.0 ± 0.7 mmol/kg_ww_, +32%, *p* = 0.03). This increase was associated with no significant change in osmolytes. A significant decrease in lactate (sham-operated: 2.0 ± 0.7, BDL: 0.9 ± 0.2 mmol/kg_ww_, -55%, *p* = 0.03) was observed for BDL rats. In addition, BDL rats displayed a significant decrease in glucose (sham-operated: 3.2 ± 0.8, BDL: 1.4 ± 0.15 mmol/kg_ww_, -56%, *p* = 0.03) and neurotransmitter γ-aminobutyric acid (GABA; sham-operated: 1.7 ± 0.2, BDL: 1.1 ± 0.2 mmol/kg_ww_, -35%, *p* = 0.02).

## Discussion

Healthy brain function requires constant and sufficient supply of oxygen and other metabolic substrates. Consequently, insufficient tissue oxygenation can have deleterious effects on all cell types and their processes, which contribute to the development of severe clinical symptoms of HE in the long-term. However, the exact pathway by which ammonia affects brain oxygenation remains unknown. In this study, we explored the effect of hyperammonaemia on brain oxygenation in the somatosensory cortex of an animal model of advanced CLD and HE. Hyperammonaemia was associated with a marked reduction in CNS tissue oxygenation, which can be considered a proxy of cerebral perfusion/blood flow at constant levels of neural activity.^37^ Although several studies have reported compromised CBF and CMRO_2_, data regarding actual brain oxygen concentration in HE have thus far been lacking.^22^

The data described herein demonstrate that preventing circulating ammonia from accumulating in BDL animals using OP, a drug known to lower systemic and brain ammonia concentration,^30^ maintains brain oxygenation within the range recorded in control animals. This clearly implicates ammonia as the driving factor responsible for the reduction of CNS perfusion. Furthermore, we also showed for the first time a reduction in other critical metabolic substrates, such as glucose and lactate, in the somatosensory cortex of BDL animals using *in vivo* ^1^H-MRS. In combination, this indicates the possibility that hyperammonaemia, seen in HE, has a detrimental impact on the supply of metabolic substrates.

A potential mechanism of how ammonia may impact brain oxygenation was described in a recent study that showed hyperammonaemia contributing to endothelial nitric oxide synthase (eNOS) downregulation through induction of inflammation and increased production of asymmetric dimethylarginine, an endogenous inhibitor of eNOS.^38^ Nitric oxide (NO) plays an important role in regulating functional microvascular perfusion^18^ and preventing vascular and endothelial dysfunction, which could contribute to HE. Correction of hyperammonaemia with OP was previously shown to restore eNOS activity resulting in improved NO metabolism.^38^

We next considered the mechanism behind the apparent reduction in metabolic substrates in the CNS as their concentration is a function of consumption and delivery. First, we asked if tissue oxygenation can be improved by agents known to increase cerebral perfusion.^39^ Indeed, BDL animals responded to increased blood concentration of CO_2_ in a manner indistinguishable from control animals with identical increases of tissue oxygen from their respective baselines. Additionally, ATZ was found to significantly increase tissue oxygenation from baseline in BDL animals, albeit not to the same level as that of controls. These observations indicate that cerebrovascular reactivity and capacity for blood vessels to dilate are intact, even during hyperammonaemia, and supply of metabolic substrates is hindered by abnormal cerebral vessel tone, thereby reducing delivery.

Evidence exists pointing towards lactate as an important energy substrate,^11^ as well as a mediator of vasodilation.^40^^,^^41^ In HE, hyperammonaemia has been associated with an impaired cortical hemichannel-mediated lactate transport, contributing to the neuronal energy deficits involved in the pathogenesis of HE.^11^ In this study, ^1^H-MRS data also revealed a significantly lower concentration of lactate, glucose, and GABA in the cortex of hyperammonaemic BDL rats, as well as elevated glutamine and decreased osmoregulatory myo-inositol and taurine concentrations (characteristic of HE^10^^,^^28^^,^^37^). Lactate has previously been shown to increase in the cerebellum of BDL rats at 8 weeks after ligation with minor changes in the hippocampus.^42^ On the other hand, brain glutamine showed the largest increase in the cerebellum and the smallest in the striatum of BDL rats using identical magnetic resonance spectroscopy measurements,^42^ confirming the already suspected brain metabolic regional difference in cirrhosis-induced HE.^10^ In parallel, decreased glucose uptake has previously been measured *ex vivo* (brain tissue)^15^ and *in vivo* ([18F]-fluorodeoxyglucose positron emission tomography [^18^F-FDG PET]^43^; plasma and cortex) using animal models similar to those used in the present study. These brain alterations indicate a dysmetabolic state and dysfunctional neurotransmission that could be arising owing to impaired delivery production and/or release of these energy substrates/neurotransmitters. Such metabolic alterations could be the cause or consequence of the reported brain hypoxia, as brain oxygenation is crucial for the production of key metabolic substrates.^44^ The combination of these factors is expected to contribute to the development of HE-associated neuropsychiatric alterations (as seen in neurodegenerative diseases^45^^,^^46^), such as memory deficits, which have previously been reported in the same animal models and at the same time point as the present recordings.^10^^,^^29^

Finally, we considered the possibility that lower MAP could be responsible for the apparent decrease in central perfusion rather than a CNS-intrinsic mechanism of altered central vessel tone. Restoring MAP by infusion of a peripheral vasoconstrictor agent successfully increased brain pO_2_ in BDL animals. This observation is in keeping with the effect of systemic vasoconstrictors on renal perfusion in patients with cirrhosis, making terlipressin or noradrenaline the drug of choice to treat hepatorenal syndrome.^47^^,^^48^ However, increasing MAP did not completely normalise the oxygenation to the same levels recorded in sham-operated controls. This suggests that hypotension is not solely responsible for the decreased brain oxygenation observed in animals with HE and central vessel tone remains a major factor in determining the supply of metabolic substrates in conditions of hyperammonaemia. Taken together, these data suggest that compromised systemic and cerebral perfusion contribute to the low brain oxygen concentration in BDL animals. The exact mechanism of the role of ammonia will need to be explored in future studies.

The limitations of the present study are that it only includes measures of tissue pO_2_ and uses this to infer changes in cerebral blood flow/perfusion. Tissue oxygenation is not solely a function of blood flow and will also be sensitive to the basal metabolic rate of oxygen. CBF measurements using functional magnetic resonance imaging (fMRI) would be the only possibility to determine brain perfusion in a way that would allow comparisons between groups of animals but were not performed in this particular study. However, CBF is known to be a significant component of brain tissue oxygenation, and we have recently shown that pO_2_ changes in the cortex of experimental animals show excellent correlation with blood oxygen level-dependent (BOLD) signals obtained using an fMRI scanner.^49^ Additionally, neuropsychological data were not collected from the subjects in the present study; however, this has been detailed in several previous publications,^10^^,^^29^ including the effect of OP on cognitive performance in the BDL model.^50^ In this study, we observe brain hypoperfusion at time points corresponding to previous reports of cognitive task performance impairment.^10^^,^^29^ We rely on the logical extension that a reduction in availability of metabolic substrates will lead to neuronal dysfunction manifesting neuropsychological impairment.^51^

Supporting the results of our study, Clément *et al.*^50^ recently demonstrated that in HE the brain, which is already compromised (decreased oxygenation and metabolic dysregulation), becomes susceptible to hypotensive insults resulting in neuronal cell death. Treating BDL rats with OP, which as we have shown here improves brain oxygenation, protected the brain against hypotension-induced neuronal cell degeneration. This provides the rationale to explore the role of drugs often used in clinical practice to increase brain perfusion, in combination with ammonia-lowering interventions, as potential therapeutic agents for treatment of HE.

In conclusion, the results presented in this study suggest that HE is associated with reduced brain tissue pO_2_ and corresponding reduction in other metabolic substrates driven by hyperammonaemia, which can be prevented with OP treatment. Although the exact mechanism of the reported phenotype is still unclear, it is proposed that ammonia could act by increasing central vascular tone, possibly via NO dysregulation. The hypoxic conditions reported in this study are sufficient to trigger astrocytic activation,^52^ as well as neuronal death,^53^ which are hypothesised to contribute to the pathogenesis of HE. This study offers the novel prospect that cerebral vascular tone could be a potential therapeutic target alongside ammonia-lowering strategies to specifically target neuronal dysfunction.

## Financial support

This study was supported by Grand Challenges UCL. AVG was supported by a Wellcome Trust Senior Research Fellowship (Ref: 200893). This study was also supported by the allocated project funding for AH from The Roger Williams Institute of Hepatology, 10.13039/100012111Foundation for Liver Research. CD was supported by the Swiss National Science Foundation under grant agreement nº 194964. The ^1^H-MRS experiments were supported by the Swiss National Science Foundation under grant agreement nº 310030_166570 and 310030_201218.

## Authors’ contributions

Study concept and design: AH, RJ, AVG, PSH. Acquisition of data: AH, PSH, CC, CK. Analysis and interpretation of data: AH, PSH, CD, DS, RJ, CC. Drafting of the manuscript: AH, CC, CK, PSH, RJ. Critical revision of the manuscript for important intellectual content: AH, RJ, PSH. Statistical analysis: AH, DS, PSH. Obtained funding: AVG, AH, RJ. Administrative, technical, or material support: AH, CK, CD, KP, AbH, PSH, DS, AVG, CC, ND. Study supervision: AH, PSH, AVG, RJ.

## Data availability statement

The data that support the findings of this study are available from the corresponding authors upon reasonable request.

## Conflicts of interest

RJ has research collaborations with Takeda and Yaqrit and consults for Yaqrit. RJ is the founder of Yaqrit Limited, which is developing UCL inventions for treatment of patients with cirrhosis. RJ is an inventor of ornithine phenylacetate, which was licensed by UCL to Mallinckrodt. He is also the inventor of Yaq-001, DIALIVE, and Yaq-005, the patents for which have been licensed by his University into a UCL spinout company, Yaqrit Ltd. All other authors report no conflict of interest.

Please refer to the accompanying ICMJE disclosure forms for further details.

## References

[1] Wijdicks E.F. (2016). Hepatic encephalopathy. N Engl J Med.

[2] Hadjihambi A., Arias N., Sheikh M., Jalan R. (2018). Hepatic encephalopathy: a critical current review. Hepatol Int.

[3] Fichet J., Mercier E., Genée O., Garot D., Legras A., Dequin P.F. (2009). Prognosis and 1-year mortality of intensive care unit patients with severe hepatic encephalopathy. J Crit Care.

[4] Kornerup L.S., Pflugrad H., Weissenborn K., Vilstrup H., Dam G. (2019). Cognitive impairment after liver transplantation: residual hepatic encephalopathy or posttransplant encephalopathy?. Hepat Med.

[5] Shawcross D.L., Shabbir S.S., Taylor N.J., Hughes R.D. (2010). Ammonia and the neutrophil in the pathogenesis of hepatic encephalopathy in cirrhosis. Hepatology.

[6] Hadjihambi A., Khetan V., Jalan R. (2014). Pharmacotherapy for hyperammonemia. Expert Opin Pharmacother.

[7] Bajaj J.S., Cordoba J., Mullen K.D., Amodio P., Shawcross D.L., Butterworth R.F. (2011). Review article: the design of clinical trials in hepatic encephalopathy – an International Society for Hepatic Encephalopathy and Nitrogen Metabolism (ISHEN) consensus statement. Aliment Pharmacol Ther.

[8] Cauli O., Rodrigo R., Llansola M., Montoliu C., Monfort P., Piedrafita B. (2009). Glutamatergic and gabaergic neurotransmission and neuronal circuits in hepatic encephalopathy. Metab Brain Dis.

[9] Hyder F., Patel A.B., Gjedde A., Rothman D.L., Behar K.L., Shulman R.G. (2006). Neuronal–glial glucose oxidation and glutamatergic-GABAergic function. J Cereb Blood Flow Metab.

[10] Braissant O., Rackayová V., Pierzchala K., Grosse J., McLin V.A., Cudalbu C. (2019). Longitudinal neurometabolic changes in the hippocampus of a rat model of chronic hepatic encephalopathy. J Hepatol.

[11] Hadjihambi A., De Chiara F., Hosford P.S., Habtetion A., Karagiannis A., Davies N. (2017). Ammonia mediates cortical hemichannel dysfunction in rodent models of chronic liver disease. Hepatology.

[12] Rao K.V., Norenberg M.D. (2001). Cerebral energy metabolism in hepatic encephalopathy and hyperammonemia. Metab Brain Dis.

[13] Attwell D., Laughlin S.B. (2001). An energy budget for signaling in the grey matter of the brain. J Cereb Blood Flow Metab.

[14] Iversen P., Mouridsen K., Hansen M.B., Jensen S.B., Sørensen M., Bak L.K. (2014). Oxidative metabolism of astrocytes is not reduced in hepatic encephalopathy: a PET study with [(11)C]acetate in humans. Front Neurosci.

[15] Dhanda S., Sunkaria A., Halder A., Sandhir R. (2018). Mitochondrial dysfunctions contribute to energy deficits in rodent model of hepatic encephalopathy. Metab Brain Dis.

[16] Heidari R. (2019). Brain mitochondria as potential therapeutic targets for managing hepatic encephalopathy. Life Sci.

[17] Willie C.K., Tzeng Y.C., Fisher J.A., Ainslie P.N. (2014). Integrative regulation of human brain blood flow. J Physiol.

[18] Hosford P.S., Gourine A.V. (2019). What is the key mediator of the neurovascular coupling response?. Neurosci Biobehav Rev.

[19] Lourenço C.F., Ledo A., Dias C., Barbosa R.M., Laranjinha J. (2015). Neurovascular and neurometabolic derailment in aging and Alzheimer's disease. Front Aging Neurosci.

[20] Iadecola C. (2017). The neurovascular unit coming of age: a journey through neurovascular coupling in health and disease. Neuron.

[21] Khot S., Tirschwell D.L. (2006). Long-term neurological complications after hypoxic-ischemic encephalopathy. Semin Neurol.

[22] Dam G., Keiding S., Munk O.L., Ott P., Vilstrup H., Bak L.K. (2013). Hepatic encephalopathy is associated with decreased cerebral oxygen metabolism and blood flow, not increased ammonia uptake. Hepatology.

[23] Iversen P., Sørensen M., Bak L.K., Waagepetersen H.S., Vafaee M.S., Borghammer P. (2009). Low cerebral oxygen consumption and blood flow in patients with cirrhosis and an acute episode of hepatic encephalopathy. Gastroenterology.

[24] Weiss N., Dam G., Rose C.F. (2018). Ammonia: this is not the end but rather the end of the beginning. J Hepatol.

[25] Jalan R., Wright G., Davies N.A., Hodges S.J. (2007). L-Ornithine phenylacetate (OP): a novel treatment for hyperammonemia and hepatic encephalopathy. Med Hypotheses.

[26] Percie du Sert N., Hurst V., Ahluwalia A., Alam S., Avey M.T., Baker M. (2020). The ARRIVE guidelines 2.0: updated guidelines for reporting animal research. PLoS Biol.

[27] Harry D., Anand R., Holt S., Davies S., Marley R., Fernando B. (1999). Increased sensitivity to endotoxemia in the bile duct-ligated cirrhotic rat. Hepatology.

[28] Rackayova V., Braissant O., McLin V.A., Berset C., Lanz B., Cudalbu C. (2016). ^1^H and ^31^P magnetic resonance spectroscopy in a rat model of chronic hepatic encephalopathy: in vivo longitudinal measurements of brain energy metabolism. Metab Brain Dis.

[29] Hadjihambi A., Harrison I.F., Costas-Rodríguez M., Vanhaecke F., Arias N., Gallego-Durán R. (2019). Impaired brain glymphatic flow in experimental hepatic encephalopathy. J Hepatol.

[30] Davies N.A., Wright G., Ytrebø L.M., Stadlbauer V., Fuskevåg O.M., Zwingmann C. (2009). L-Ornithine and phenylacetate synergistically produce sustained reduction in ammonia and brain water in cirrhotic rats. Hepatology.

[31] Hosford P.S., Christie I.N., Niranjan A., Aziz Q., Anderson N., Ang R. (2018). A critical role for the ATP-sensitive potassium channel subunit K_IR_ 6.1 in the control of cerebral blood flow. J Cereb Blood Flow Metab.

[32] Nizari S., Basalay M., Chapman P., Korte N., Korsak A., Christie I.N. (2021). Glucagon-like peptide-1 (GLP-1) receptor activation dilates cerebral arterioles, increases cerebral blood flow, and mediates remote (pre)conditioning neuroprotection against ischaemic stroke. Basic Res Cardiol.

[33] Haase C.G., Becka M., Kuhlmann J., Wensing G. (2005). Influences of caffeine, acetazolamide and cognitive stimulation on cerebral blood flow velocities. Prog Neuropsychopharmacol Biol Psychiatry.

[34] Vorstrup S., Henriksen L., Paulson O.B. (1984). Effect of acetazolamide on cerebral blood flow and cerebral metabolic rate for oxygen. J Clin Invest.

[35] Theparambil S.M., Hosford P.S., Ruminot I., Kopach O., Reynolds J.R., Sandoval P.Y. (2020). Astrocytes regulate brain extracellular pH via a neuronal activity-dependent bicarbonate shuttle. Nat Commun.

[36] Estrela H.F., Damasio E.S., Fonseca E.K., Bergamaschi C.T., Campos R.R. (2016). Differential sympathetic vasomotor activation induced by liver cirrhosis in rats. PLoS One.

[37] Leithner C., Royl G. (2014). The oxygen paradox of neurovascular coupling. J Cereb Blood Flow Metab.

[38] Balasubramaniyan V., Wright G., Sharma V., Davies N.A., Sharifi Y., Habtesion A. (2012). Ammonia reduction with ornithine phenylacetate restores brain eNOS activity via the DDAH-ADMA pathway in bile duct-ligated cirrhotic rats. Am J Physiol Gastrointest Liver Physiol.

[39] Hoiland R.L., Fisher J.A., Ainslie P.N. (2019). Regulation of the cerebral circulation by arterial carbon dioxide. Compr Physiol.

[40] Hein T.W., Xu W., Kuo L. (2006). Dilation of retinal arterioles in response to lactate: role of nitric oxide, guanylyl cyclase, and ATP-sensitive potassium channels. Invest Ophthalmol Vis Sci.

[41] Gordon G.R., Choi H.B., Rungta R.L., Ellis-Davies G.C., MacVicar B.A. (2008). Brain metabolism dictates the polarity of astrocyte control over arterioles. Nature.

[42] Simicic D., Pierzchala K., Rackayovà V., Braissant O., Mitrea S., Sessa D. (2019). 33 in vivo longitudinal 1H MRS study of hippocampal, cerebral and striatal metabolic changes in the adult brain using an animal model of chronic hepatic encephalopathy. Am J Gastroenterol.

[43] Mosso J., Yin T., Poitry-Yamate C., Simicic D., Lepore M., McLin V.A. (2022). PET CMR_glc_ mapping and ^1^H MRS show altered glucose uptake and neurometabolic profiles in BDL rats. Anal Biochem.

[44] Watts M.E., Pocock R., Claudianos C. (2018). Brain energy and oxygen metabolism: emerging role in normal function and disease. Front Mol Neurosci.

[45] Silverman D.H., Small G.W., Chang C.Y., Lu C.S., Kung De Aburto M.A., Chen W. (2001). Positron emission tomography in evaluation of dementia: regional brain metabolism and long-term outcome. JAMA.

[46] Zhou C., Huang Y., Przedborski S. (2008). Oxidative stress in Parkinson's disease: a mechanism of pathogenic and therapeutic significance. Ann N Y Acad Sci.

[47] Hadengue A., Gadano A., Moreau R., Giostra E., Durand F., Valla D. (1998). Beneficial effects of the 2-day administration of terlipressin in patients with cirrhosis and hepatorenal syndrome. J Hepatol.

[48] Ginès P., Schrier R.W. (2009). Renal failure in cirrhosis. N Engl J Med.

[49] Hosford P.S., Wells J.A., Christie I.N., Lythgoe M.F., Millar J., Gourine A.V. (2019). Electrochemical carbon fiber-based technique for simultaneous recordings of brain tissue PO_2_, pH, and extracellular field potentials. Biosens Bioelectron X.

[50] Clément M.A., Bosoi C.R., Oliveira M.M., Tremblay M., Bémeur C., Rose C.F. (2021). Bile-duct ligation renders the brain susceptible to hypotension-induced neuronal degeneration: implications of ammonia. J Neurochem.

[51] Gibson G.E., Pulsinelli W., Blass J.P., Duffy T.E. (1981). Brain dysfunction in mild to moderate hypoxia. Am J Med.

[52] Angelova P.R., Kasymov V., Christie I., Sheikhbahaei S., Turovsky E., Marina N. (2015). Functional oxygen sensitivity of astrocytes. J Neurosci.

[53] Banasiak K.J., Haddad G.G. (1998). Hypoxia-induced apoptosis: effect of hypoxic severity and role of p53 in neuronal cell death. Brain Res.

