# Supplementary material for: Abnormal brain oxygen homeostasis in an animal model of liver disease
Source: JHEP Rep. 2022 May 24;4(8):100509. doi: 10.1016/j.jhepr.2022.100509 (PMC9293761; doi:10.1016/j.jhepr.2022.100509)
Supplement: Multimedia component 1 [file mmc1.pdf]

Abnormal brain oxygen homeostasis in an animal model of liver disease

Anna Hadjihambi, Cristina Cudalbu, Katarzyna Pierzchala, Dunja Simicic, Chris Donnelly, Christos Konstantinou, Nathan Davies, Abeba Habtesion, Alexander V. Gourine, Rajiv Jalan, Patrick S. Hosford

Table of contents

Fig. S1 2

Table S1 2

Table S2 3

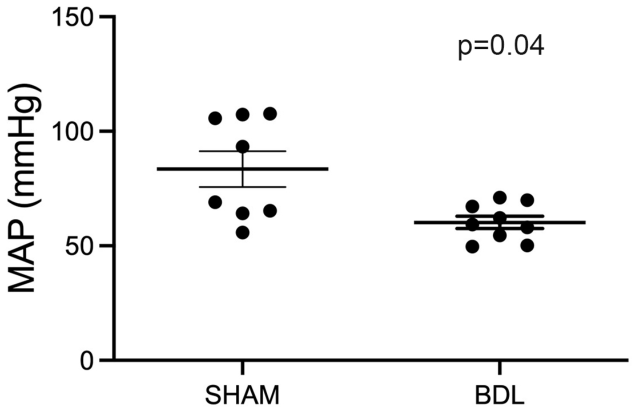

Fig. S1. Mean arterial pressure measured in an animal model of HE. Data expressed as mean±SEM and compared using Mann Whitney test. MAP: Mean arterial pressure.

Parameters	*Sham*	*BDL*	*BDL+OP*	*Sham+OP*
**Ammonia, µmol/L**	56±3	141±1**	61±0.5	50±5
**Albumin, g/L**	35±4	23±0.2**	23±1**	30±2
**Total protein, g/L**	50±0.3	38±0.3**	42±0.5*	53±1
**Bilirubin, µmol/L**	5±0.5	185±5**	163±2**	6±0.5
**ALT, U/L**	11±0.4	130±0.5**	86±1**	13±0.5

Table S1. Plasma biochemistry of experimental animal groups. ALT: alanine aminotransferase. Data expressed as mean±SEM and compared to the sham group using one-way ANOVA. **$p<0.001$, *$p<0.05$

	Sprague-Dawley		*Wistar*	
**Parameters**	**Sham**	**BDL**	**Sham**	**BDL**
**Ammonia, µmol/L**	#56±3	#141±1**	##10±1	##49±16*
**Bilirubin, µmol/L**	$5±0.5	149±10**	<9	$$137±9**

Table S2. Ammonia and bilirubin concentrations of the two different animal models of hepatic encephalopathy. Data expressed as mean±SEM and compared to the sham group of each strain, using student t-test. **p<0.001, *p<0.05. #Plasma was measured using Cobas Integra II system (Roche Diagnostics). ##Whole fresh blood was measured using PocketChemTM (BA PA-4140). $Plasma was measured using Cobas Integra II system (Roche Diagnostics). $$ Plasma was measured using Reflotron® Plus system (F. Hoffmann-La Roche Ltd). For bilirubin measurements one-way ANOVA was used to compare all four groups. No significant differences were observed between the two BDL groups.
