# Supplementary material for: Abnormal brain oxygen homeostasis in an animal model of liver disease
Source: JHEP Rep. 2022 May 24;4(8):100509. doi: 10.1016/j.jhepr.2022.100509 (PMC9293761; doi:10.1016/j.jhepr.2022.100509)
Supplement: Multimedia component 2 [file mmc2.docx]

**Journal of Hepatology**

**CTAT methods**

Tables for a “Complete, Transparent, Accurate and Timely account” (CTAT) are now mandatory for all revised submissions. The aim is to enhance the reproducibility of methods.

- Only include the parts relevant to your study
- Refer to the CTAT in the main text as ‘Supplementary CTAT Table’
- Do not add subheadings
- Add as many rows as needed to include all information
- Only include one item per row

**If the CTAT form is not relevant to your study, please outline the reasons why:**

|  |
| --- |

- 1. **Antibodies**

| **Name** | **Citation** | **Supplier** | **Cat no.** | **Clone no.** |
| --- | --- | --- | --- | --- |
|  |  |  |  |  |

- 1. **Cell lines**

| **Name** | **Citation** | **Supplier** | **Cat no.** | **Passage no.** | **Authentication test method** |
| --- | --- | --- | --- | --- | --- |
|  |  |  |  |  |  |

- 1. **Organisms**

| **Name** | **Citation** | **Supplier** | **Strain** | **Sex** | **Age** | **Overall n number** |
| --- | --- | --- | --- | --- | --- | --- |
| Rat |  | Charles Rivers Laboratories, Inc | Sprague-Dawley | **Male** | **6-8 weeks** | **85** |
| Rat |  | Charles Rivers Laboratories, Inc | Wistar | **Male** | **6-8 weeks** | **14** |

- 1. **Sequence based reagents**

| **Name** | **Sequence** | **Supplier** |
| --- | --- | --- |
|  |  |  |

- 1. **Biological samples**

| **Description** | **Source** | **Identifier** |
| --- | --- | --- |
|  |  |  |

- 1. **Deposited data**

| **Name of repository** | **Identifier** | **Link** |
| --- | --- | --- |
|  |  |  |

- 1. **Software**

| **Software name** | **Manufacturer** | **Version** |
| --- | --- | --- |
| Spike 2 | **Cambridge Electronic Design** | **7.20** |
| Prism | **GraphPad** | **9.2** |

- 1. **Other (e.g. drugs, proteins, vectors etc.)**

| L-ornithine and phenylacetate (OP) OCR-002 | Ocera Therapeutics, CA, USA | **Donation** |
| --- | --- | --- |
| Optical fluorescence PO2 Monitor | Oxylite^TM^, Oxford Optronics |  |
| 9.4 T MRI System | Varian/Magnex Scientific |  |
| Acetazolamide | Sigma Aldrich | **Cat. A6011** |
| Phenylephrine | Sigma Aldrich | **Cat. P1240000** |

- 1. **Please provide the details of the corresponding methods author for the manuscript:**

| **Patrick Steven Hosford**  **Current Address: Brain Science Centre, RIKEN, Wako, Saitama, Japan**  **p.hosford@ucl.ac.uk** |
| --- |

**2.0 Please confirm for randomised controlled trials all versions of the clinical protocol are included in the submission. These will be published online as supplementary information.**

| **Not applicable** |
| --- |
